# Edible Bird's Nest Attenuates Neuroinflammation and Cognitive Impairment in a Model of Chronic Fatigue Syndrome

**DOI:** 10.1002/fsn3.71685

**Published:** 2026-04-25

**Authors:** Xinyuan Wang, Dongliang Wang, Yi Chai, Yijia Zhang, Huihui Wang, Taiqi Qu, Wenrui Zhang, Man Yuan, Shihan Gao, Yanan Sun, Yixuan Li

**Affiliations:** ^1^ State Key Laboratory of Food Nutrition and Safety, College of Food Science and Engineering Tianjin University of Science and Technology Tianjin China; ^2^ Hebei Edible Bird's Nest Fresh Stew Technology Innovation Center Langfang China; ^3^ Key Laboratory of Functional Dairy, co‐Constructed by Ministry of Education and Beijing Municipality, College of Food Science &Nutritional Engineering China Agricultural University Beijing China; ^4^ Beijing Advanced Innovation Center for Food Nutrition and Human Health, Department of Nutrition and Health China Agricultural University Beijing China

**Keywords:** chronic fatigue syndrome, edible birds nest, microglia, neuroinflammation, Val‐Trp

## Abstract

Chronic fatigue syndrome (CFS) is a debilitating disorder characterized by persistent fatigue with an unclear pathophysiology. Increasing evidence suggests that neuroinflammation plays an important role in the development of CFS; however, the underlying mechanisms remain poorly defined. In this study, we evaluated the effects of edible bird's nest (EBN) in a sleep‐deprivation–induced CFS‐like mouse model. EBN treatment improved motor and cognitive performance, reduced neuronal necrosis, and preserved synaptic structure in the prefrontal cortex. Blood metabolomic profiling revealed elevated levels of the dipeptide valyl‐tryptophan (Val‐Trp) following EBN administration. Functional assays showed that Val‐Trp attenuates neuroinflammation by suppressing nuclear factor kappa‐B signaling, thereby shifting microglia from a pro‐inflammatory to a homeostatic state. These findings implicate Val‐Trp as a candidate bioactive metabolite associated with the neuroprotective effects of EBN, providing preclinical evidence to support exploration of natural‐product‐derived interventions for CFS.

## Graphical abstract

Edible bird's nest (EBN) alleviates chronic fatigue syndrome (CFS)‐like symptoms in a sleep‐deprivation–induced mice model by suppressing neuroinflammation and improving neurobehavioral performance. EBN administration enhances motor and cognitive functions, reduces neuronal necrosis, and preserves synaptic integrity in the prefrontal cortex. Metabolomic analysis identifies a significant increase in the circulating dipeptide valyl‐tryptophan (Val‐Trp) following EBN treatment. Mechanistically, Val‐Trp attenuates neuroinflammation by inhibiting NF‐κB signaling and promoting the phenotypic shift of microglia from a pro‐inflammatory (M1‐like) state to a homeostatic phenotype. In vitro assays using LPS‐stimulated microglia further confirm the anti‐inflammatory role of Val‐Trp. Collectively, these findings reveal Val‐Trp as a key bioactive metabolite mediating the neuroprotective effects of EBN, highlighting a potential natural‐product‐derived therapeutic strategy for CFS.

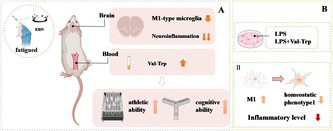

## 1 Introduction

Chronic Fatigue Syndrome (CFS) is a medical condition characterized by persistent fatigue lasting at least 6 months (Lim and Son 2020). It concurrently impairs limb mobility, cognitive function, and emotional stability. The clinical phenotype is defined by substantial reduction in both physical and cognitive capacities. Commonly reported symptoms include head heaviness, depressive symptoms, and pain in the extremities (Sandler and Lloyd 2020). Research on CFS has primarily focused on immune mechanisms, psychological factors, and viral triggers—with immune regulation being most extensively studied (Bjørklund et al. 2020). Fatigue and sleep disorders are widespread in modern society, and many patients with CFS also experience significant sleep disturbances (Mariman et al. 2013). Experimental findings indicate a strong correlation between sleep deprivation and increased fatigue. The average course of CFS is 10 years, and drug therapies can cause adverse effects. This underscores the need for treatments that minimize adverse effects while ensuring therapeutic efficacy. The pathogenesis of CFS is multifactorial, involving neuroendocrine, hormonal, metabolic, immune, psychological, social, and infectious elements. Elevated levels of pro‐inflammatory factors in the blood of patients with CFS are common (Giloteaux et al. 2023). Neuroinflammation in the brains of patients with CFS has been observed through brain positron emission tomography (PET) imaging (Nakatomi et al. 2014). Microglia, the brain's resident macrophages, play a crucial role in immunoregulation by clearing cellular debris and damaged neurons. Chronic neuroinflammation can cause aberrant microglial activation, driving their shift to the pro‐inflammatory (M1‐like), which further exacerbates neuroinflammatory and neurotoxic cascades. Such processes are closely tied to neurodegenerative diseases, suggesting that restoring microglial homeostasis can benefit both CFS and neurodegenerative conditions (Nam et al. 2018). Returning microglia from the M1 state to a resting phenotype is vital for balancing inflammatory mediators, reprogramming cellular metabolism, and modulating key signaling pathways. Activation of NF‐κB and MAPK in M1‐like microglial phenotype drives inflammation; thus, inhibiting NF‐κB or blocking MAPK phosphorylation can reduce pro‐inflammatory factor release and help restore resting function.

Edible bird's nest (EBN), secreted by the *Aerodramus* genus predominantly in Southeast Asia (Kong et al. 2022), contains high levels of polyunsaturated fatty acids (PUFAs), sialic acids, and essential amino acids (Haghani et al. 2016). Proteins constitute ~63% of its nutritional profile, indicating its potential as a source of bioactive peptides. Recent studies have identified EBN‐derived peptides exhibiting antioxidant and anti‐inflammatory activities (Lee et al. 2024). Two pentapeptides within its low‐molecular‐weight fraction have been proposed as potential anti‐fatigue agents. EBN is widely consumed as a traditional food product, and available evidence suggests a favorable safety profile; however, systematic clinical safety evaluations remain limited.

To investigate the therapeutic potential of EBN in mitigating CFS‐related symptoms, we assessed its effects in a murine model of CFS induced by sustained sleep deprivation. We evaluated behavioral and neuropathological variations, analyzed blood metabolomic profiles to identify candidate bioactive molecules, and validated mechanistic actions in BV2 microglia. The objective was to determine whether EBN and its metabolites could mitigate CFS‐like symptoms and to provide a foundation for developing natural product‐based interventions for the disorder.

## 2 Materials and Methods

### 2.1 Animals

Sixty female BALB/c mice aged 7 months (Spearfish [Beijing] Biotechnology Co. Ltd.), as ME/CFS shows higher prevalence in females (Vahratian et al. 2023), each weighing 30 ± 3 g, were used in this study. They were housed under controlled environmental conditions (temperature: 22°C ± 1°C, and light/dark cycle: 12/12 h) and given standard chow and ad libitum water during a 1‐week acclimation period before the experiment. All procedures followed the Guide for the Care and Use of Laboratory Animals and were approved by the ethics committee of China Agricultural University (reference number GSCS‐2025‐02‐001).

### 2.2 CFS Model

Sixty female BALB/c mice were randomly allocated to five groups (*n* = 12 per group): Control, Model, Model + EBN‐L, Model + EBN‐M, and Model + EBN‐H. Mice were allocated at *n* = 12/group to account for potential attrition during the prolonged sleep‐deprivation protocol. Based on an a priori analysis plan and consistency with prior studies, six mice per group were randomly selected for each primary endpoint (behavioral tests, cytokines/hormones, histology, and metabolomics), and these samples were used for statistical analyses (reported as *n* = 6 in figure legends). Remaining animals were maintained as backups and were not analyzed unless needed to replace excluded samples. The maximum dosage referenced from prior research ensured methodological consistency (Duong et al. 2022). EBN was administered by oral gavage at doses of 0.3, 0.45, or 0.6 g/kg body weight per day, selected based on a recent murine study reporting effective and well‐tolerated oral EBN doses of 0.285–0.649 g/kg (Li, Xu, et al. 2024). To aid translational interpretation, we further estimated human‐equivalent doses (HED) using body‐surface‐area scaling (mouse Km = 3; human Km = 37) (Reagan‐Shaw et al. 2008), yielding ~0.024–0.049 g/kg/day (i.e., ~1.5–2.9 g/day for a 60‐kg adult), which is within the range of customary dietary consumption reported for EBN products (Kim et al. 2022). The CFS model was induced via severe sleep deprivation. Sleep deprivation was implemented using a modified protocol described (Zhang et al. 2022), comprising six cycles of 48 h deprivation followed by 24 h recovery. Sleep deprivation was implemented using a modified rotary rod method. Food and water were available ad libitum throughout. Model validity was confirmed using forced running, grip strength, open‐field, and Y‐maze tests performed the day after modeling.

### 2.3 Preparation of EBN

Dried edible bird's nest (EBN) material was commercially obtained from Hebei Edible Bird's Nest Fresh Stew Technology Innovation Center, sourced from nests originally imported from Indonesia. The raw EBN samples were initially rinsed thoroughly with running tap water to remove surface impurities and residual feathers. After air‐drying under ambient laboratory conditions, precisely 5 g of cleaned EBN was weighed and mixed with 95 mL of ultrapure water (Milli‐Q grade) in a heat‐resistant flask (Bi et al. 2024). The mixture was subsequently boiled at 95°C for 15 min to ensure complete dissolution.

### 2.4 Behavioral Assessment

Behavioral tests were conducted the day after modeling to confirm the validity of the CFS model. Peripheral fatigue was evaluated using forced running and grip strength tests, while central fatigue was assessed with open‐field and Y‐maze tests.

#### 2.4.1 Forced Running Test

The forced running test was conducted in accordance with established guidelines for accuracy (Garrigos et al. 2021). Mice underwent a 3‐day training period before testing. Training consisted of running on a treadmill (Zhongshi Technology, Beijing) at 10 m/min, three times daily with an interval of more than 2 h between each training session (Sato et al. 2020). During formal testing, the running speed was increased by 1 m/min every 5 min, starting at 6 m/min. The test was terminated when a mouse failed to return to the treadmill or remained on the electric grid for > 10 s. Total running time was recorded, after which the mouse was transferred to a stress‐free environment for recovery.

#### 2.4.2 Grip Strength Tests

To assess the motor skills of mice, the grip strength test was conducted (Lv et al. 2023). The apparatus was positioned on a horizontal platform. When a mouse grasped the horizontal bar with all four limbs, its tail was gently pulled backward until grip release occurred. Each mouse underwent five trials, and the mean value was recorded.

#### 2.4.3 Open‐Field Test

To evaluate the mice's exploratory behavior and anxiety levels, the open‐field test was performed (Dong et al. 2020). Mice were placed in a quiet, dimly lit environment, and the apparatus was cleaned with a 75% ethanol solution between each trial. Each mouse was placed in the open‐field apparatus and allowed to move freely for 5 min while being monitored. ANY‐Maze software was used to analyze the center dwell time (the arena was divided into 16 squares, with the central 4 squares defined as the center area) and the total movement distance.

#### 2.4.4 Y‐Maze Test

Working memory was assessed by the spontaneous alternation task in a Y‐maze, in accordance with established methods. (Dong et al. 2020). Mice were placed at a designated starting point in the maze and allowed to explore the arms freely for a brief period. Entry into an arm was defined as all four limbs being within it. Alternation was defined as successive entries into all three arms. The number of arm entries and alternations was recorded to calculate the percentage of correct alternation behavior.

### 2.5 Sample Collection

After behavioral testing, all animals rested for 1 day. Mice were fasted for 12 h before sacrifice, with the final gavage given before fasting. Blood samples were collected from the orbital sinus under isoflurane anesthesia, and serum was obtained by centrifugation at 4000 rpm for 15 min. Serum samples were stored at −80°C. Subsequently, the heart was perfused with saline, and the brain (whole brain and prefrontal cortex), tibialis anterior (TA), and soleus (SOL) muscles were rapidly dissected and frozen at −80°C.

### 2.6 Measurement of Biochemical Indicators

Serum IL‐6, IL‐1β, TNF‐α, cortisol, and 5‐HT were quantified using commercially available mouse ELISA kits (Shanghai Enzyme Science and Technology Co. Ltd.; IL‐6 Cat# [MK5737A], IL‐1β Cat# [MK2776A], TNF‐α Cat# [MK2868A], cortisol Cat# [MK3301A], 5‐HT Cat# [MK3179A]). All assays were performed according to the manufacturer's instructions. 2.7 Hematoxylin and eosin (H&E) staining.

Transverse sections were taken at the largest cross‐sectional area of the TA. Brain tissues, including the prefrontal cortex, were prepared for coronal sectioning. Samples were fixed in 4% paraformaldehyde (Beijing Solarbio Science & Technology Co. Ltd.), processed for hematoxylin–eosin staining, and imaged under a microscope for morphological analysis.

### 2.7 Golgi Staining

Whole brains were immersed in Golgi fixative and maintained in darkness for 7 days, with daily fixative replacement. After fixation, tissues were rinsed in running water for 24 h until pale yellow. Samples were then impregnated with silver nitrate, transferred to developing solution, and gently agitated until neuronal structure became distinct. Tissue samples were dehydrated through a graded ethanol series, cleared in xylene for 24 h, and embedded in paraffin. Sections were treated with ammonia silver drops for 5 min, rinsed with distilled water, dehydrated again with graded ethanol, sealed, and scanned using Pannoramic Scanner software for detailed analysis.

### 2.8 Blood Metabolome

Untargeted serum metabolomics was performed using a UHPLC–MS/MS system (Thermo Fisher Scientific Q Exactive Orbitrap) equipped with a Hypersil GOLD C18 column (100 × 2.1 mm, 1.9 μm). Chromatographic separation was achieved at 40°C with a flow rate of 0.3 mL/min using mobile phases of 0.1% formic acid in water (A) and 0.1% formic acid in acetonitrile (B). Analyses were conducted in both positive and negative electrospray ionization modes.

Raw data were processed with Compound Discoverer 3.1 for peak detection, alignment, and normalization. Features with relative standard deviation > 30% in quality‐control samples were removed, and signal intensities were log_2_‐transformed and Pareto‐scaled before statistical analysis. Metabolite identification was based on accurate mass (±5 ppm) and MS/MS fragment matching against public databases (HMDB, KEGG, mzCloud), following Metabolomics Standards Initiative level 2 criteria.

### 2.9 Cell Culture

BV2 microglial cells were obtained from Wuhan Zishan Biological Co. Ltd. (Cat# [STCC20009P]) and routinely tested to be mycoplasma‐negative. Cells were maintained in BV2 Cell‐Specific Culture Medium (Cat# [GZ20009‐500 mL]) at 37°C in a humidified incubator with 5% CO_2_. Cells were passaged at 70%–80% confluence.

### 2.10 Western Blotting

BV2 microglial cells were seeded into six‐well plates. Lipopolysaccharide (LPS; Beijing Solarbio Science & Technology Co. Ltd.) at 1 μg/mL was added to the model group to induce inflammation. The treatment group received an additional 1 μL/mL of the dipeptide Val‐Trp. After 24 h, cells were lysed in RIPA buffer for protein extraction. Protein content was quantified using a bicinchoninic acid (BCA) kit (Beijing Solarbio Science & Technology Co. Ltd.). Membranes were blocked with 5% bovine serum albumin for 1 h, incubated overnight at 4°C with the following primary antibodies (all from Proteintech Group Inc., Wuhan): GAPDH (Cat# 60004–1‐Ig, 1:5000), NFκB‐p65 (Cat# 10745–1‐AP, 1:1000), P‐NFκB‐p65 (Cat# 3033–1‐AP, 1:1000), and iNOS (Cat# 18985–1‐AP, 1:1000), washed with PBST, incubated with secondary antibodies for 1 h, washed again, and developed.

### 2.11 Determination of Nitric Oxide (NO) Concentration

BV2 microglial cells were seeded into 96‐well plates. Inflammation was induced by adding 1 μg/mL of LPS to the model group. The treatment group also received 1 μL/mL of Val‐Trp. After 24 h, NO levels were measured using a commercial assay kit (Beyotime Biotechnology, Cat# [S0021S]).

### 2.12 Data Analysis

Data are expressed as mean ± SD. Normality was assessed using the Shapiro–Wilk test and homogeneity of variance using Levene's test. Differences among groups were analyzed by one‐way ANOVA followed by Tukey's post hoc test., using IBM SPSS Statistics and GraphPad Prism v10.0.0. A *p* < 0.05 was considered significant.

## 3 Results

### 3.1 Body Weight and Organ Index

**FIGURE 1:**
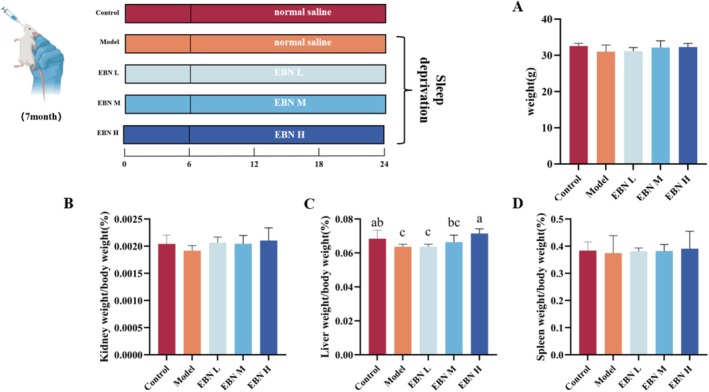
Body weight and organ indices: (A) Body weight; (B) spleen index; (C) lung index; (D) kidney index. Data are expressed as mean ± SD (*n* = 6). Statistical analysis was performed using one‐way ANOVA followed by Tukey's multiple‐comparison test; data are presented as mean ± SD (*n* = 6 per group). Differences were considered statistically significant at *p* < 0.05.

To evaluate whether bird's nest (EBN) had any adverse effects on mice, we assessed body weight and organ indices. Results showed no significant differences in body weight, kidney index, or spleen index between groups. In contrast, the model group exhibited a marked reduction in liver index, which was restored to near‐normal levels following EBN treatment (Figure 1A–D). This recovery is closely linked to immune regulation, suggesting that EBN may play a role in modulating immune function (Van Braeckel‐Budimir et al. 2016). Overall, compared with the control group, EBN supplementation did not induce significant changes in the basic physiological indicators of mice.

### 3.2 EBN Improves Symptoms of Cognitive and Working Memory Decline due to CFS

**FIGURE 2:**
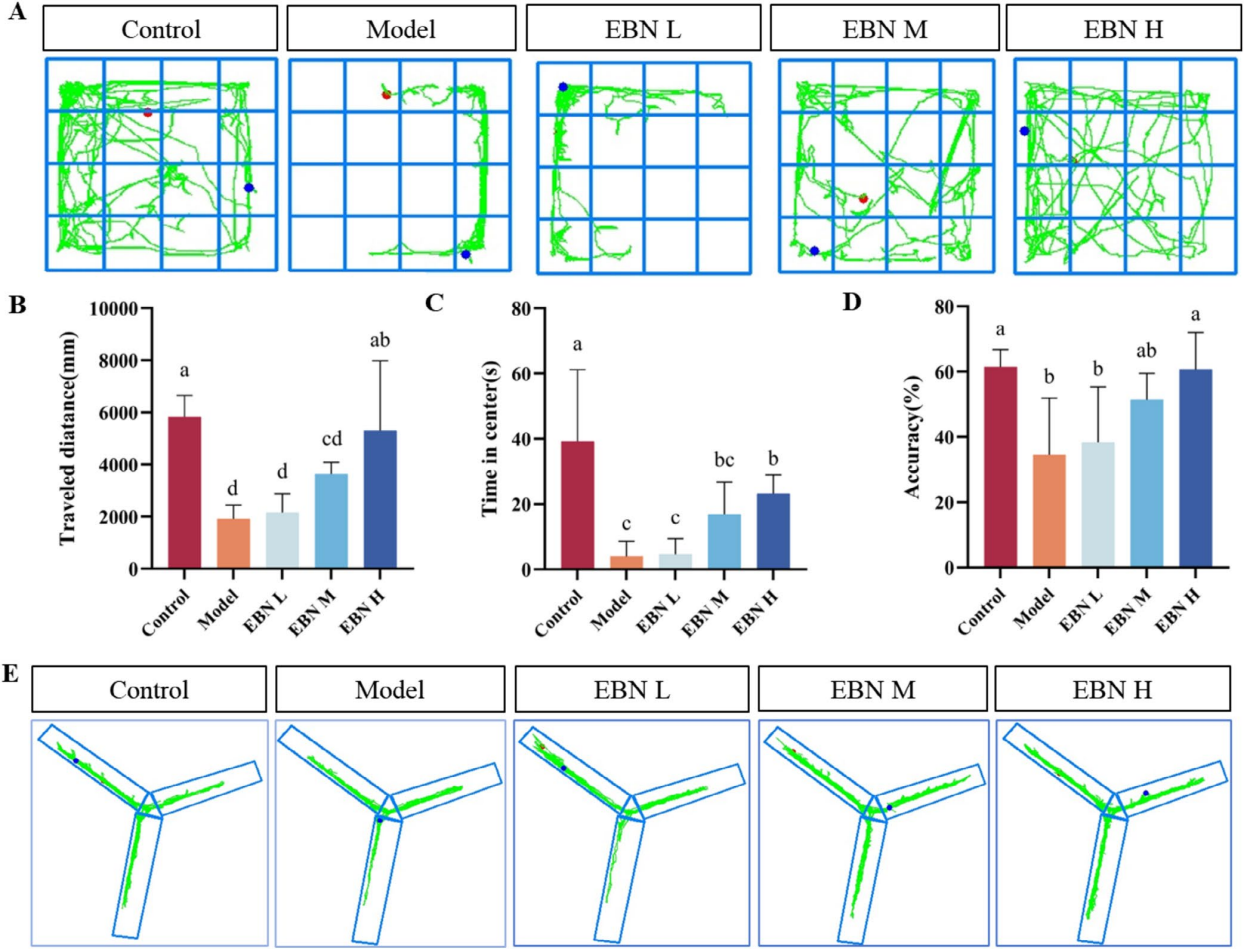
EBN alleviates cognitive and working memory decline associated with CFS. (A) Movement path in the open field (*n* = 6); (B) total movement distance in the open‐field test; (C) duration spent in the central area; (D) accuracy of spontaneous alternation in the Y‐maze test; (E) movement path in the Y maze. Statistical analysis was performed using one‐way ANOVA followed by Tukey's multiple‐comparison test; data are presented as mean ± SD (*n* = 6 per group). Differences were considered statistically significant at *p* < 0.05.

CFS affects cognitive ability, working memory, and emotional status. The open‐field test is widely used to assess anxiety levels and exploratory behavior (Shaw et al. 2007). Figure 2A shows the path diagram of mice moving in the open field. The total distance traveled by the model group was significantly less than that of the control group, indicating reduced exploratory drive due to CFS. The EBN‐H group traveled 1.8‐fold farther than the Model group, whereas the EBN‐M group showed a 0.9‐fold increase. The EBN‐L group exhibited a non‐significant upward trend. These findings indicate that EBN treatment restores exploratory behavior in a dose‐dependent manner (Figure 2B). In the open field, the central area—considered a danger zone—is typically avoided by mice with high anxiety. The Model group spent significantly less time in the center compared with the Control group, while the EBN‐H group spent 5.8‐fold longer in the center than the Model group (Figure 2C). The Y‐maze spontaneous alternation test was used to assess working memory (Kraeuter et al. 2019). Figure 2D illustrates the correct rate of spontaneous alternations, while Figure 2D illustrates the paths of the mice moving in the Y maze. The model group displayed a significant decline in spontaneous alternation frequency compared with controls. The spontaneous alternation accuracy of the EBN‐H group was 0.8‐fold higher than that of the Model group and reached 99% of the control level. These results indicate that EBN treatment effectively restores working memory in mice to near‐normal levels.

### 3.3 EBN Increases Exercise Capacity and Reduces Muscle Damage in CFS Models

**FIGURE 3:**
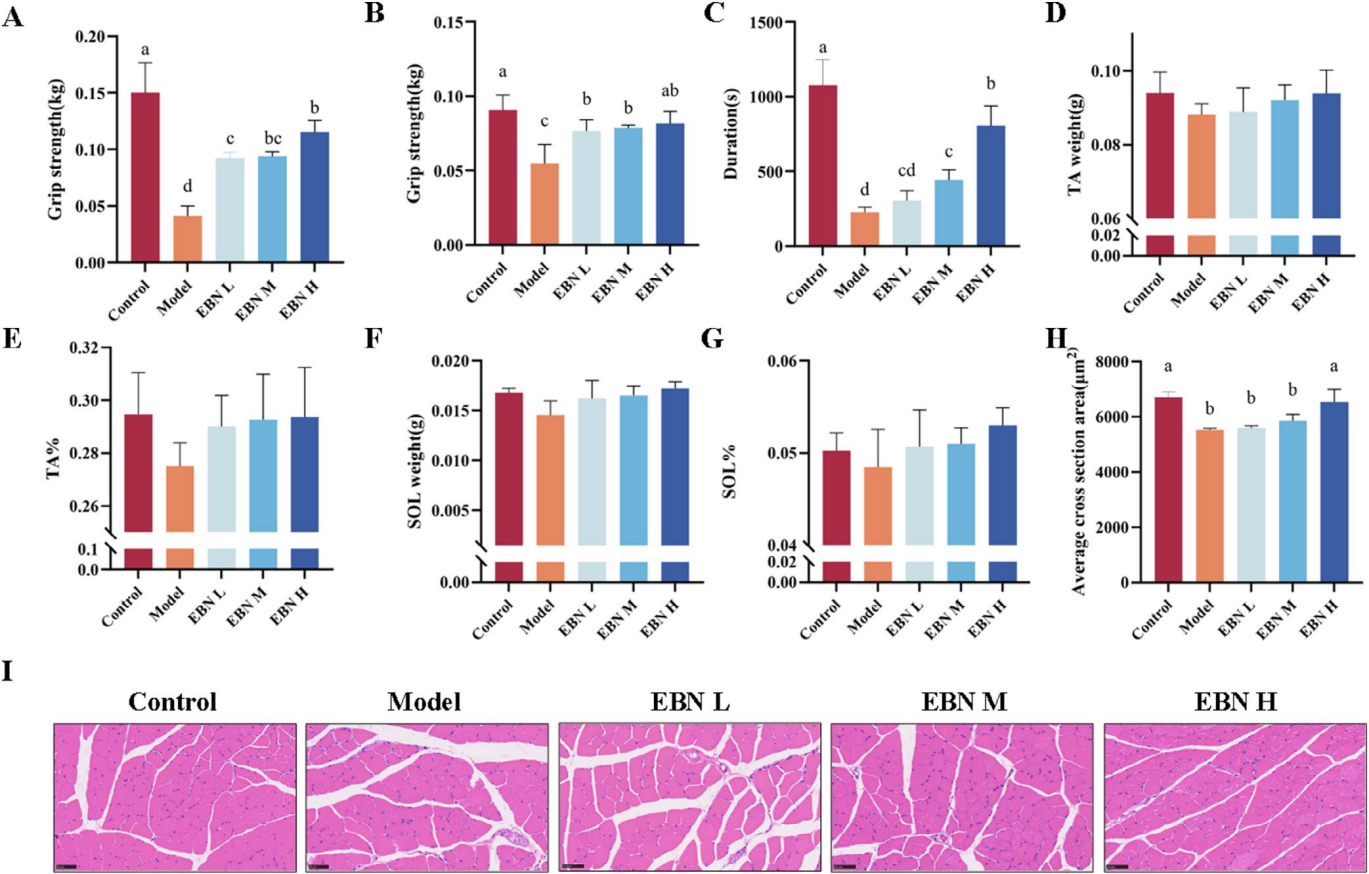
Muscle strength and locomotor activity of mice (A) Hind paw gripping force (B), forepaw gripping force (C), duration of forced running (D) Tibialis anterior weight (E) Tibialis anterior weight ratio (F) Soleus weight (G) Soleus weight ratio (H) TA muscle fiber area (I) Tibialis anterior cross‐section HE staining pattern. Statistical analysis was performed using one‐way ANOVA followed by Tukey's multiple‐comparison test; data are presented as mean ± SD (*n* = 6 per group). Differences were considered statistically significant at *p* < 0.05.

Patients with CFS often report that even mild physical exertion precipitates debilitating fatigue, with sleep providing no relief (Gerwyn and Maes 2017). To evaluate locomotor capacity, fore‐ and hind‐paw grip strength and forced running were measured in mice (Figure 3A–C). The forced running duration in the EBN‐H group was 3.6‐fold longer than those in the model group. Likewise, fore‐ and hind‐paw grip strengths in the EBN‐H group were 1.5‐fold and 2.8‐fold higher, respectively, than those in the model group. Both metrices were significantly reduced in the model group compared to controls, indicating that EBN markedly improves motor performance in mice. Many patients with CFS experience generalized muscle weakness and exercise intolerance (Garner and Baraniuk 2019). To investigate muscular changes, tibialis anterior (TA) and soleus (SOL) muscle weights were assessed (Figure 3D–G). Although the model group exhibited lower TA and SOL weight ratios, the differences were not statistically significant. EBN treatment increased these ratios compared with the model group, demonstrating notable improvement compared with controls. Furthermore, HE staining revealed a reduced TA muscle cross‐sectional area in the Model group (Figure 3H,I), which was largely restored by high‐dose EBN treatment. Under EBN‐H treatment, the cross‐sectional area reached 98% of the control value, indicating near‐complete recovery.

### 3.4 EBN Regulates Dysregulated Hormones, Neurotransmitters, and Pro‐Inflammatory Factors and Reduces Brain Tissue Damage

**FIGURE 4:**
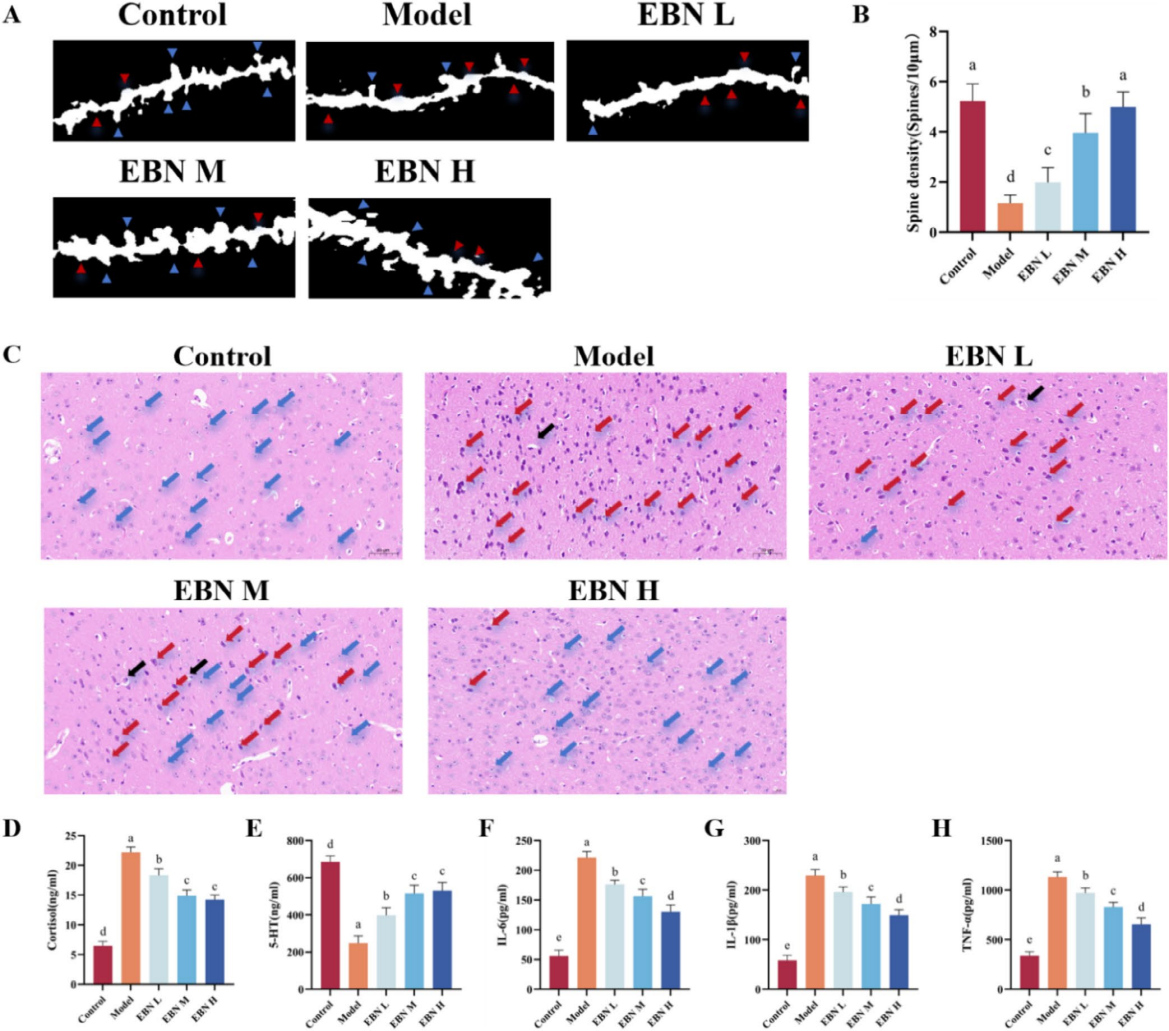
EBN alleviated inflammation and reduced chronic fatigue syndrome (CFS)‐induced neuronal damage in the brain. (A) Golgi staining of the prefrontal cortex (B) Density map of dendritic spines in the prefrontal cortex (*n* = 6) (C) HE staining of the prefrontal cortex (D) Cortisol content (E) 5‐HT (F) Interleukin‐6 content (G) Interleukin‐1β content (H) Tumor necrosis factor‐α content. Statistical analysis was performed using one‐way ANOVA followed by Tukey's multiple‐comparison test; data are presented as mean ± SD (*n* = 6 per group). Differences were considered statistically significant at *p* < 0.05.

Neurodegenerative diseases often cause neuronal damage or reduced synaptic ridge density in the brain, and extensive neuronal necrosis typically triggers neuroinflammation (Omdal 2020). Damage to specific brain regions results in distinct symptoms, with many CFS studies focusing on the prefrontal cortex (Brain‐Regional Characteristics and Neuroinflammation in ME/CFS Patients From Neuroimaging: A Systematic Review and Meta‐Analysis–PubMed, n.d.)—a key area for movement control and executive decision‐making, closely linked to CFS. To assess the therapeutic effects of bird's nest on the brain, we conducted Golgi staining of the prefrontal cortex, the region most affected by CFS. Figure 4A presents the synaptic cristae map of prefrontal cortex neurons, where blue arrows mark intact synaptic cristae and red arrows indicate marked loss, which was notably reduced in CFS. The density of synaptic cristae in the EBN H group was 3.3 times higher than that in the Model group, suggesting that EBN treatment restores synaptic cristae density—crucial for cognitive function (Figure 4B). CFS, a chronic disorder involving neuroimmune dysfunction, is associated with prefrontal cortex neuroinflammation, as demonstrated by neuroimaging studies (Cleare et al. 2005). In the control group, the prefrontal cortex appeared structurally normal, with evenly distributed, healthy neurons (blue arrows), whereas the model group exhibited extensive neuronal necrosis and swelling. The highest EBN concentration produced the most pronounced therapeutic benefit (Figure 4C).

Chronic fatigue results in dysregulation of hormones, neurotransmitters, and pro‐inflammatory mediators. Elevated cortisol probably contributes to chronic inflammation (Knezevic et al. 2023). Cortisol, a hormone closely linked to mood, was significantly increased in the model group but decreased in the high‐dose bird's nest group (Figure 4D). Reduced serotonin (5‐HT) can cause sleep disturbances and may lead to anxiety or depression, ultimately impairing cognitive performance (Ursin 2002). In the model group, 5‐HT levels were significantly low, while EBN treatment demonstrated a significant improvement (Figure 4E). These findings indicate that EBN can help correct hormone and neurotransmitter imbalances in patients with CFS.

Pro‐inflammatory cytokines—including interleukin‐6 (IL‐6), interleukin‐1β (IL‐1β), and tumor necrosis factor‐α (TNF‐α)—are upregulated in patients with CFS (Maksoud et al. 2023). These mediators can cross the blood–brain barrier or activate vagal signaling, contributing to chronic low‐grade inflammation and accelerating neurodegeneration. All three pro‐inflammatory factors were significantly upregulated in the model group, while EBN treatment reduced them in a dose‐dependent manner (Figure 4F–H). Collectively, these results, along with brain pathology observations, were associated with CFS—consistent with other research—which intensifies inflammation. However, EBN administration markedly attenuates brain tissue damage and inflammatory responses.

### 3.5 Bioactive Peptides With Increased Levels in the Blood After EBN Use May Be Key Substances in Alleviating CFS

**FIGURE 5:**
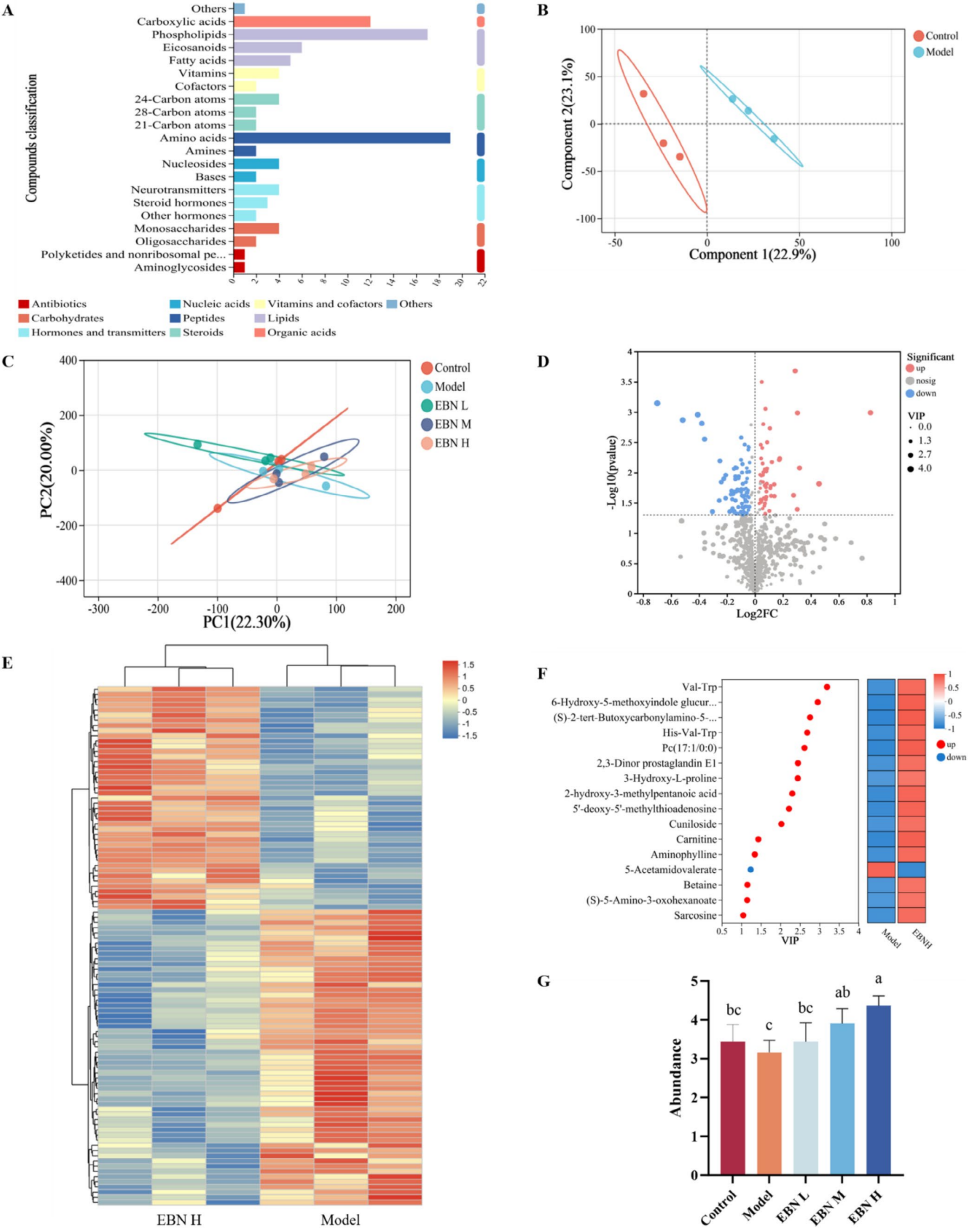
Blood metabolome analysis included several key components: (A) Histogram of Kyoto Encyclopedia of Genes and Genomes (KEGG) compound classification statistics. (B) Partial least squares discriminant analysis (PLS‐DA) scores comparing the control group with the model group. (C) Principal component analysis (PCA). (D) Differential volcano plots for the EBN H group versus the model group. (E) Clustering heatmap for the EBN H group versus the model group. (F) Variable importance in projection (VIP) plots for the EBN H group versus the model group. (G) Screened differential metabolites for the EBN H group versus the model group. Statistical analysis was performed using one‐way ANOVA followed by Tukey's multiple‐comparison test; data are presented as mean ± SD (*n* = 6 per group). Differences were considered statistically significant at *p* < 0.05.

Studies have shown that blood metabolites can cross into the brain (Varma et al. 2018). In our study, the most abundant metabolite classes across all groups were amino acids, phospholipids, and carboxylic acids (Figure 5A). Partial least squares discriminant analysis (PLS‐DA) score plots revealed clear separation between the Control and Model groups, indicating a strong classification effect from the experimental intervention (Figure 5B). In contrast, principal component analysis (PCA) showed only minor variability between groups, likely because PCA is more sensitive to large‐scale differences and less responsive to subtle micronutrient changes (Figure 5C).

To further identify potential metabolites that alleviate CFS symptoms, we screened for substances whose levels increased or decreased. Comparing the EBN H group with the model group, we found 51 metabolites upregulated and 81 downregulated in the EBN H group (Figure 5D). The variable importance in projection (VIP) plot showed that the most prominently upregulated compounds were mainly peptides or amino acid–related metabolites (Figure 5F). This suggests that oligopeptides or amino acids are likely responsible for the relief of CFS symptoms. Notably, the abundance of valine–tryptophan (Val‐Trp) increased by 38% compared with the model group (Figure 5G), and the rise in Val‐Trp levels in the blood associated with EBN was dose‐dependent. In summary, Val‐Trp emerged as a prominently upregulated dipeptide following EBN administration and may represent a candidate mediator associated with the observed phenotypic improvements.

### 3.6 The Dipeptide Val‐Trp Alleviates Neuroinflammation in BV2 Microglia

**FIGURE 6:**
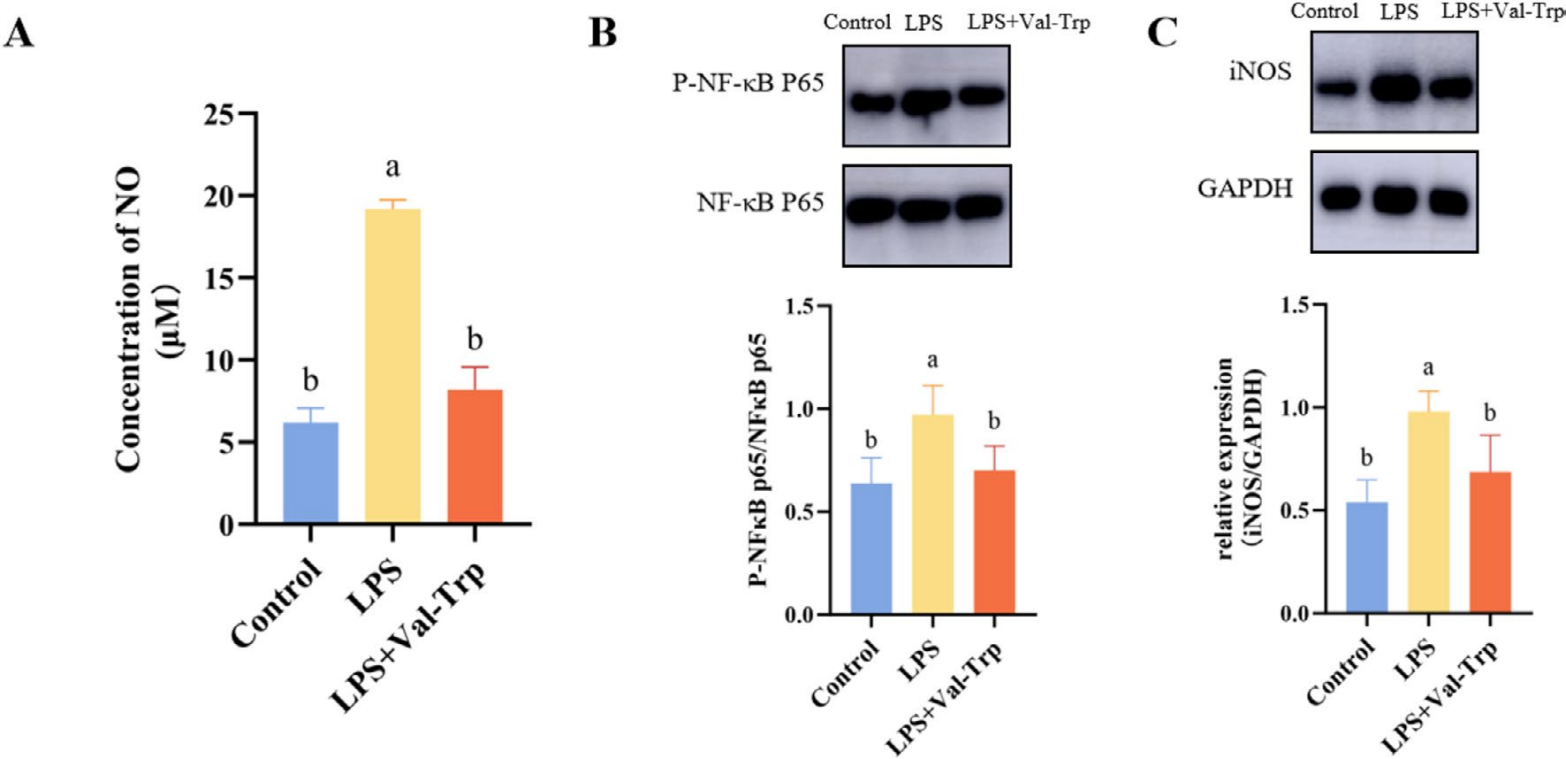
EBN treatment elevates the dipeptide Val‐Trp, which alleviates neuroinflammation in the BV2 microglial cell model. (A) Nitric oxide (NO) concentration (B) Protein blot analysis of NF‐κB p65 and phosphorylated NF‐κB p65 (C) Protein blot analysis of iNOS and GAPDH. Statistical analysis was performed using one‐way ANOVA followed by Tukey's multiple‐comparison test; data are presented as mean ± SD. Differences were considered statistically significant at *p* < 0.05.

When the brain is exposed to a pro‐inflammatory microenvironment, microglia are activated into the M1‐like microglial phenotype, which exacerbates neuroinflammation (Long et al. 2024). This inflammatory state leads to neuronal necrosis, impairing both motor function and cognitive abilities. NO concentration is commonly used to assess neuroinflammation in brain tissue (Tewari et al. 2021). In an LPS‐induced neuroinflammation model, NO levels were significantly higher in the Model group compared with the control group. However, treatment with the dipeptide Val‐Trp markedly reduced NO content; after Val‐Trp administration, NO levels decreased by 57% relative to the LPS group and were close to those of the Control group (Figure 6A). At the signaling level, neuroinflammation was accompanied by marked activation of the NF‐κB pathway (Li, Dai, et al. 2024) Phosphorylated NF‐κB p65 expression was strongly increased in the model group but suppressed following Val‐Trp treatment (Figure 6B), indicating that Val‐Trp reduces neuroinflammation by inhibiting NF‐κB activation. In parallel, Val‐Trp modulated microglial polarization: expression of iNOS, a canonical M1‐like microglial phenotype marker, was significantly elevated in the model group but reduced after Val‐Trp administration (Figure 6C). In summary, these results suggest that Val‐Trp alleviates neuroinflammation by suppressing NF‐κB activation and limiting M1‐like microglial phenotype polarization, thereby providing a potential mechanistic basis for its neuroprotective effects.

## 4 Discussion

Our results show that EBN administration was associated with improvements in behavioral performance and reduced inflammatory readouts in a sleep‐deprivation–induced CFS‐like mouse model. EBN administration was associated with improved cognitive and motor performance, accompanied by reduced histopathological injury in the prefrontal cortex and a shift toward lower peripheral cytokine levels. In BV2 microglia, Val‐Trp was observed to suppress NF‐κB pathway activation and was associated with reduced expression of M1‐like markers under LPS stimulation. These findings are consistent with the possibility that EBN may ameliorate CFS‐like phenotypes through modulation of immune–neural interactions in this model.

Neuroimaging and biomarker studies have implicated neuroinflammation and altered network dynamics in CFS (Steiner et al. 2023), particularly within prefrontal circuits that are involved in the regulation of fatigue and cognition (Nakatomi et al. 2014). Our histological data provide structural evidence of prefrontal injury in the model, with lower injury scores/spine loss observed in EBN‐treated mice. The association between inflammatory readouts and behavioral impairment suggests that EBN‐derived small molecules could influence neuroimmune processes relevant to phenotype expression in this model.

Previous research has demonstrated that metabolomic analyses have identified 1365 metabolites involved in brain–blood interactions, suggesting that substances can be exchanged between the brain and blood (Wang et al. 2025). Among these, Val‐Trp emerged as a candidate mediator in our study. Peptides under 500 Da are often considered potentially BBB‐permeable; however, the BBB penetration of Val‐Trp remains hypothetical and requires experimental confirmation. Our data are consistent with a plausible peripheral mechanism that may indirectly influence central neuroinflammation. Val‐Trp attenuated NF‐κB activation in microglia, steering them away from a pro‐inflammatory state. EBN administration was associated with elevated circulating Val‐Trp in a dose‐dependent manner, which may contribute to attenuated microglial activation and preserved synaptic integrity. Collectively, these findings support a conceptual model in which EBN‐derived Val‐Trp exerts neuroprotective effects consistent with improved cognitive and motor performance in CFS‐like mice. This integrated evidence highlights the potential of natural‐product‐derived metabolites as candidates for therapeutic development targeting neuroimmune dysregulation in CFS.

This study has several limitations. First, the sleep‐deprivation paradigm models key fatigue‐related and neuroinflammatory features but does not recapitulate the full clinical heterogeneity of ME/CFS, which may limit direct clinical extrapolation. Second, Val‐Trp was quantified in serum but not directly in brain tissue; therefore, its blood–brain barrier transport and central bioavailability remain unverified. Third, the untargeted metabolomics findings are correlative and do not establish causality between Val‐Trp and the observed behavioral/neuropathological improvements. Finally, our study focused on female mice and a single treatment window and thus may not capture sex‐dependent or time‐dependent effects. Future studies will include targeted quantification of Val‐Trp in the brain, BBB permeability assays, controlled Val‐Trp supplementation/neutralization experiments, and longer‐term assessments to determine durability and safety of the effects.

## Author Contributions

**Dongliang Wang:** funding acquisition. **Man Yuan:** project administration. **Yijia Zhang:** conceptualization. **Xinyuan Wang:** writing – original draft, software, validation. **Yanan Sun:** writing – review and editing. **Taiqi Qu:** methodology. **Shihan Gao:** resources. **Yi Chai:** methodology. **Wenrui Zhang:** investigation. **Huihui Wang:** data curation. **Yixuan Li:** visualization, supervision.

## Funding

This research was funded by the 111 project from the Education Ministry of China (B18053).

## Conflicts of Interest

The authors declare no conflicts of interest.

## Data Availability

The data that support the findings of this study are available from the corresponding author upon reasonable request.
